# Personality and interest in general practice: results from an online survey among medical students

**DOI:** 10.1186/s12875-024-02682-0

**Published:** 2024-12-12

**Authors:** Maike Krauthausen, Tobias Leutritz, Martin J. Koch, Pamina E. Hagen, Sarah König, Anne Simmenroth

**Affiliations:** 1 Department of General Practice, University Hospital Würzburg, Würzburg, Germany; 2 Institute for Medical Teaching and Medical Education Research, University Hospital Würzburg, Würzburg, Germany

**Keywords:** Medical studies, Students, Personality, General Practice/Family Medicine, ‘Big five’, Career choice

## Abstract

**Background:**

The growing shortage of General Practitioners (GPs) is a Europe-wide challenge, particularly in rural areas. In Germany, the situation is worsened by an ageing workforce of GPs and insufficient training of new doctors. Many newly qualified physicians choose careers outside primary care or prefer to work part-time to balance work and family life. To address this problem, it is essential to understand the factors that influence medical students’ specialty choice, and then to take action to encourage them to specialise in General Practice (GP). In addition to medical school experiences, rural placements, or characteristics of the specialty, personality traits have been shown to influence students’ specialty decisions. A well-researched approach to assessing personality is the Five-Factor Model, which measures personality on the dimensions *openness* (to experience), *conscientiousness*, *extraversion*, *agreeableness*, and *neuroticism*. Using the findings about the links between students’ personalities and an increased interest in a career in General Practice may be an approach to raising the number of GPs.

**Objectives:**

We aimed to examine how students’ personality traits influence their interest in General Practice and their current intention to pursue General Practice as a specialty.

**Methods:**

In March 2021, we started an ongoing online survey among medical students at the University of Würzburg and assessed cross-sectional data about the ‘Big Five’ personality traits and aspects of career choice. Until December 2022, we invited three cohorts of first-year beginners, and one cohort each of third-, fifth- and sixth-year students via email to participate in the survey. For statistical analysis, we performed linear regression and extended it into a path model to examine the relationship between students’ personality traits, their interest in General Practice, and whether they would currently choose General Practice for their future specialty. We controlled for possible confounding effects of age, gender, and current semester by using covariates.

**Results:**

Higher levels of *agreeableness* and *neuroticism* predicted greater interest in GPs, whereas higher levels of *conscientiousness* and *openness* predicted less interest in GPs. The effects of *extraversion* were unclear. Age was a significant predictor of interest, with older age associated with greater interest in General Practice. Gender was not a significant predictor of interest in General Practice, and the results for semester were inconclusive. The interest in General Practice is a predictor of the intention to choose GP as a specialty. The personality dimensions show an indirect predictive effect on the intention to choose GP, mediated by interest in GP. In total, R² = 7.7% of the variance of the interest in GP was explained by the combination of personality dimensions and covariates.

**Conclusion:**

Our study reveals that students’ personality traits predict their interest in General Practice and their intention to choose it as a specialty. Personality assessments can be integrated into counselling services to help students better understand their traits. Our findings highlight the great potential of considering personality in career counselling during medical education or even the extent of admission criteria to medical school by personality-related criteria.

**Supplementary Information:**

The online version contains supplementary material available at 10.1186/s12875-024-02682-0.

## Introduction

The shortage of General Practitioners (GPs) is a well-known problem that remains a challenge, and the reasons for this lack are complex. While the rapidly growing ageing population worldwide continues to increase the demand for primary care, every third of GPs currently working in Germany is 60 years old or older [1]. At the same time, not enough new doctors are being trained to meet the demand, as the newly qualified physicians in Germany tend to work outside the healthcare sector more often and choose other fields of care than GP more frequently [2–4]. In contrast to the past, more than 60% of German GP trainees are female [5]. When they pursue a GP career, many of them are working part-time to seek a better work-life balance or to balance family and career. Both young female and male GPs tend to work as employed doctors in existing practices without economic responsibility [6]. Overall, projections indicate an increasing shortage of over 10,500 primary care doctors by 2030 in Germany, especially in rural areas [7].

To address this challenge in the long term, it is essential to encourage more students to specialise in General Practice and to prioritise their training as future GPs. In this context, it is important to understand which factors influence the interest in General Practice. A review of existing literature offers a range of different factors influencing medical students’ choice of specialty: during medical school, the curricula, curricula, experiences during placements, and in-depth programmes influence the decision of a specialty [8, 9]. Further, for example, GP is a preferential selection of students who grew up in rural areas, and the immersion of medical students in the rural environment at different stages of their medical studies for a longer period has proven to be an effective tool to increase their interest in GP [10, 11]. Besides, the perceived attractiveness of a particular specialty and aspects of quality of life, such as work-life balance and family friendliness, are described to have an increasing impact [12, 13]. Initially, most students show little stability in their preference for a specialty and become more confident in their choice towards the end of their studies [14–16].

Finally, the importance of students’ personality is discussed. In this context, personality is perceived as an individual’s relatively consistent and persistent disposition manifesting in patterns of thoughts, feelings, and behaviours [17, 18]. There are well-researched approaches to measure personality, like the Five-Factor model or ‘Big Five’ model of personality that focuses on the dimensions *openness (to experience)*, *conscientiousness*, *extraversion*, *agreeableness*, and *neuroticism* [19]. Differences in personality are influenced by age and gender across cultures [20, 21]. For instance, women reported themselves to be higher in *agreeableness* than men. Regarding age, changes in personality levels have been described especially during young adulthood, before personality traits tend to gain more stability with increasing age [21]. Within certain limits, changes in one’s personality occur in reaction to life events or through repeated new learning experiences, for example as part of targeted (therapeutic) interventions like psychotherapy [22–24].

Regarding the influence of personality on students’ specialty choice, many studies have assessed personality traits and their influence on career choices in general [25] and specialty choices of medical students [18, 26–31] in detail. Most of them aim to identify distinct patterns of personality that predict the choice of a specialty, or vice versa, try to describe favourable personality traits for different specialties. For instance, specializing in General Practice is associated with higher *agreeableness*, showing sympathetic, trusting, and cooperative behaviour, while lower *agreeableness* was associated with specializing in surgery [19, 28, 29].

Our study aims to contribute to approaches that investigate the role of students’ personalities in their interest in GP to increase the number of future GPs. We assessed personality traits in medical students from different years of study and explored the association between their personality, their basic interest in General Practice, and whether they would currently choose General Practice as a specialty. We expected that students show outlined manifestations of personality traits that influence their interest in General Practice and their current specialty choice in this field.

## Methods and material

### Study design and setting

This study was part of a longitudinal monitoring of medical students at Julius-Maximilians-University Würzburg, Germany, regarding interrelationships between students’ study admission, study success, and specialty choice.

### Survey

The following description of our survey is in line with the ‘Checklist for reporting results of internet E-surveys’ (CHERRIES) [32].

### Design, approval, and informed consent process

The survey comprised items concerning students’ demography, study permission, influences on choosing medical studies, and aspects of the study process. Approval for the study was granted directly by the Ethics Committee at the University of Würzburg. However, the data protection officer was involved and students consented to the study and data management right at the beginning of the survey. Voluntary register numbers for longitudinal tracking cannot be combined with personal data by study personnel.

### Development and pre-testing

After implementing all questions within the survey platform EvaSys^®^ (version 8.0, Electric Paper Evaluationssysteme GmbH, Lüneburg, Germany), which is hosted by the university, technical feasibility was tested as well as a prognosis of the required time to fill the questionnaire. A test trial running from 22/3/2021 to 15/4/2021 with the first cohort led to minor adjustments of some items, e.g., linguistic issues or the number of response categories. Questions regarding the presented data were not affected. Thus, we decided to include this data as well. The first wave of the survey started in May 2021 and continued (see Table 1 for an overview of surveys).

**Table 1:** Characteristics of the survey and sample

Year	Data collection period	Invited (*N*)	Response (*n*)	Age (M, SD)	Gender (*n* m/f)	Interest in GP° (M, SD)	Current Choice of GP (*n*, %)
1 (start)	22/03/-15/04/2021	170	83	21.5 (2.8)	25/57	3.5 (1.2)	18 (22.2)
1 (start)	17/05/-16/06/2021	167	76	22.0 (4.0)	18/58	2.8 (1.4)	2 (2.7)
1 (start)	02/11/-25/11/2021	177	90	20.5 (2.5)	20/70	3.2 (1.3)	19 (22.4)
1 (start)	14/11/-13/12/2022	164	84	20.9 (3.0)	30/54	3.0 (1.4)	9 (10.8)
3 (start)	06/05/-31/05/2021	157	69	23.3 (3.5)	25/43^+^	3.0 (1.3)	5 (7.8)
5 (end)	26/05/-28/06/2021	143	66	26.0 (3.2)	24/41*	3.2 (1.4)	11 (16.9)
6 (end)	13/09/-18/10/2021	153	84	27.3 (2.3)	25/58^+^	3.1 (1.3)	10 (12.8)
6 (end)	01/03/-15/04/2022	153	80	28.0 (2.8)	21/59	2.9 (1.4)	7 (8.9)
	**Total**	1284	628	23.4 (4.2)	188/440	3.1 (1.3)	81 (13.3)

*1 student refers to ‘diverse’; ^+^1 student chose ‘not specified’; **°** item ‘How likely would you choose General Practice as a specialty?’, ranging from 1=‘very unlikely’ to 5=‘very likely’

### Recruitment process and selection of study subjects

The survey was designed to capture crucial points in time during medical studies, which takes six years in Germany. We addressed the first year, representing the very beginning of all studies. After the first state examination (end of 2nd year), medical students in Germany start their clinical section, so we chose this period to address students’ characteristics at the beginning of their clinical education. The fifth year ends with a second state examination, followed by a year of internship. Therefore, we chose this year to address students’ characteristics before experiencing education outside the university, working in a hospital, or ambulatory care. Addressing sixth-year students, we wanted to assess characteristics at the end of the entire study programme.

### Survey administration

All students were contacted via email with a link to the closed online survey (see Supplement 1 for wording). We used reminder emails to increase the return rate and offered free mugs with the logo of the faculty for each participant. The exact time frame for each survey is given in Table 1.

There was no randomization in the item presentation, but distinct filtering to present only appropriate items, e.g. skipping questions about doctoral theses for first-semester students. Overall, a maximum of 152 items was presented to the students. Only fully completed questionnaires could be submitted, including the answers ‘not specified’ for those not willing to answer a specific question. For open questions, the response was not mandatory and could be left empty. Reviewing the entries before final submission was possible.

### Response rates and preventing multiple entries from the same individual

As only completed questionnaires could be submitted, the presented response rate in Table 1 is the completion rate, whereas participation rates might be higher. Survey links were randomly personalized by the survey system to ensure single participation.

### Analysis

Handling of incomplete surveys was not applicable. As we only receive timestamps from completion, the exclusion of surveys that were filled in too fast was not feasible. No statistical correction was taken into account. Only implausible years of birth were set to N/A.

### Study variables

Students were asked to report the likelihood of choosing General Practice as their field of specialty after finishing medical school on a five-point Likert scale (1=‘not likely at all’ – 5=‘very likely’), referred to as ‘Interest in General Practice’ as the first outcome variable. The second outcome variable was the ‘Intention to choose General Practice’ (for specialty), which was operationalised by students’ current first specialty choice (nominal data level). To assess personality traits, we used the German short version of the Big Five Inventory (BFI-K) to assess different personality traits in medical students by self-evaluation [33]. It consists of 21 items rated on a five-point Likert scale to measure the ‘Big Five’ personality factors: *openness*, *conscientiousness*, *extraversion*, *agreeableness*, and *neuroticism* [34]. We used this short version of the personality questionnaire to limit the extent of items in the survey. To ensure the reliability of the dimensions of the ‘Big Five’ scales, we calculated Cronbach’s α as a measure for internal consistency which was (still) acceptable for all scales. More information on the internal consistency of personality assessment is provided in the supplement of this article (see Supplement 2).

### Data analysis

**Fig. 1:**
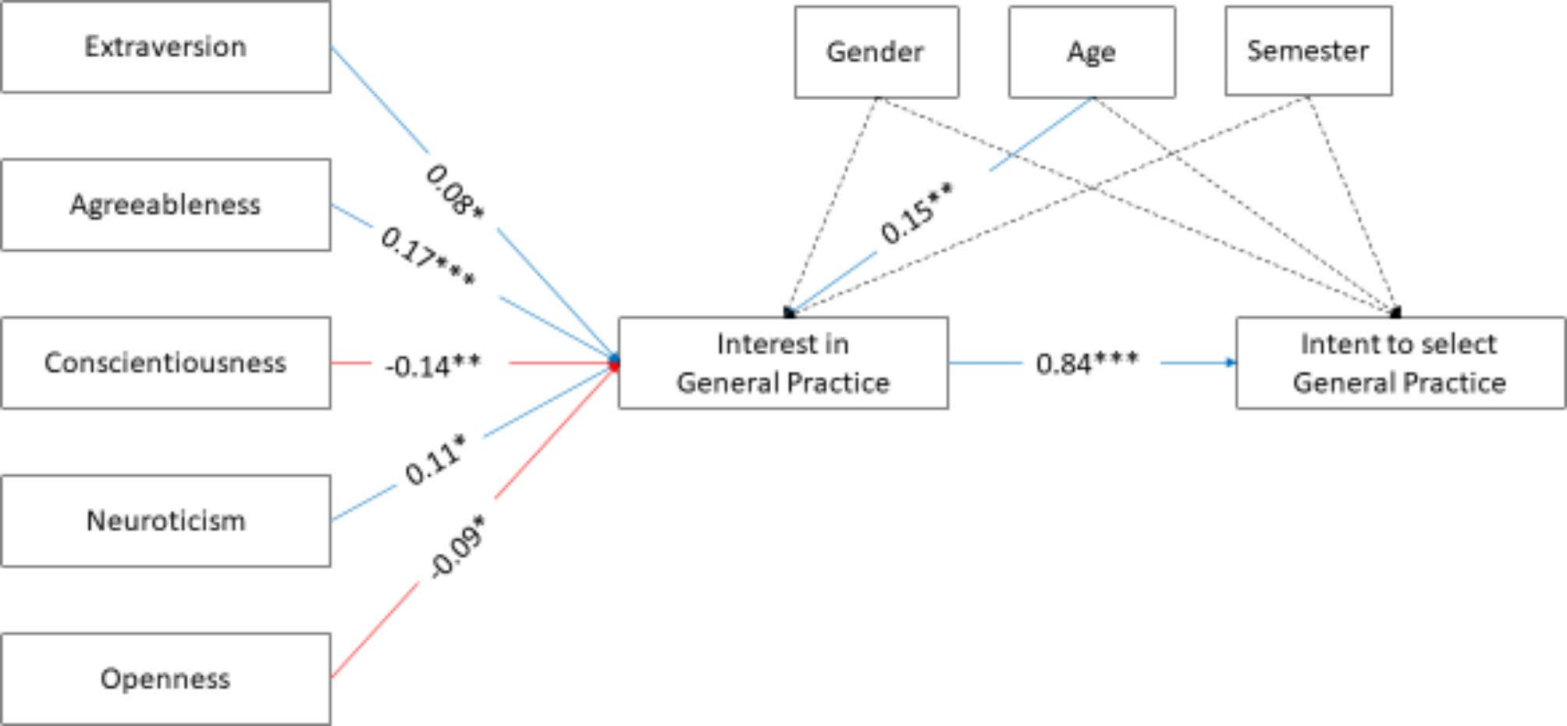
Path model of the mediation effects of the personality dimensions on the intent mediated by the interest in GP. Legend Fig. 1: Asterisks indicate the significance of the linear effects (* *p* < .05, ** *p* < .01, *** *p* < .001). Dashed lines indicate non-significant effects. Red arrows indicate negative effects, and blue arrows indicate positive effects. Numbers indicate standardized effect sizes of the linear effects

In a first step, a multiple linear regression was calculated using IBM SPSS version 26 [35]. We used the ‘Big Five’ personality dimensions and the covariates gender, age, and semester to predict the interest in GP. In a second step, we added the intention to choose GP to the model as a second-level outcome variable predicted only by the interest in GP and the covariates. The path model was tested using a diagonally weighted least squares-path analysis with the package lavaan version 0.6.17 [36] with R version 4.3.3 [37]. In addition to the effects of the linear regression from the first analysis (effects of personality and socio-demographics on interest in GP), we included the effect of interest in GP on the intention to become a General Practitioner (see Fig. 1). We controlled for the effects of the covariates age, gender, and semester on the intention and estimated the indirect effects of the personality traits on the intention mediated by the interest in GP.

### Data management and data protection

In the preparation of the study, the data protection officer of the University of Würzburg was consulted. In compliance with the European General Data Protection Regulation (GDPR), written informed consent was obtained. All data were collected and processed anonymously. To ensure anonymity, only the year of birth was inquired. The collected data are retained by the Office of Student Affairs and will be deleted 10 years after finishing the overall study in which the survey was conducted.

### Ethics

The study was approved on 8 March 2021 by the Ethics Committee at the University of Würzburg, Germany (ref. no 2021011803).

## Results

### Sample characteristics

Out of the 1284 students who were invited to participate in the survey, 628 responded, resulting in a response rate of 48.9% (for more details, see Table 1).

In this sample, the average age was 23.4 years (*SD* = 4.2) and the gender distribution was 70.1% female and 29.9% male. One person selected the category ‘diverse’ and was excluded from data analysis, as the cell population was too small here and gender effects needed to be addressed. Regarding the interest in General Practice, 46.6% of the students indicated an interest in choosing General Practice (by answering ‘likely’ or ‘very likely’ when asked how likely they would choose General Practice as a specialty). Addressing the intention to choose General Practice (current first choice of specialty), 13.9% of the entire sample quoted General Practice as their current first choice for specialty, including students who already committed themselves to becoming a General Practitioner due to study permission or sponsorship. The average characteristics of the five personality dimensions are shown in Table 2.

**Table 2:** Sample characteristics on the ‘Big five’ personality dimensions related to semester and interest in General Practice

Year*	Interest in GP (M, SD)	Current choice of GP (*n*, %)	Neuroticism (M, SD)	Openness (M, SD)	Agree-ableness (M, SD)	Conscien-tiousness (M, SD)	Extra-version (M, SD)
1 (start)	3.1 (1.3)	48 (14.8)	2.8 (0.8)	3.8 (0.7)	3.5 (0.8)	4.1 (0.6)	3.8 (0.8)
3 (start)	3.0 (1.3)	5 (7.8)	2.8 (0.8)	3.9 (0.8)	3.5 (0.9)	4.2 (0.6)	3.9 (0.8)
5 (end)	3.2 (1.4)	11 (16.9)	2.6 (0.8)	3.7 (0.8)	3.7 (0.7)	4.1 (0.5)	3.6 (0.9)
6 (end)	3.0 (1.3)	17 (10.8)	2.9 (0.8)	3.7 (0.7)	3.5 (0.7)	4.1 (0.5)	3.6 (0.8)
Total	3.1 (1.3)	81 (13.3)	2.8 (0.8)	3.8 (0.7)	3.5 (0.8)	4.1 (0.6)	3.7 (0.8)

*summarised from different data collection periods as presented in Table 1

### Prediction of interest in general practice

The statistical requirements for the linear regression were tested and considered to be met (for detailed information, see Supplement 2).

In total, *R²* = 7.7% of the variance of the interest in General Practice was explained by the combination of personality dimensions and covariates. This was a significant proportion of variance (*F*(8, 607) = 6.21, *p* < .001). *Agreeableness*, *conscientiousness*, *neuroticism*, and *openness* were significant predictors of interest in General Practice (see Table 3). Higher values of *agreeableness* and *neuroticism* predicted higher values of interest in GP when holding the respective other variables constant, while higher values of *conscientiousness* and *openness* indicated lower interest in General Practice (see Table 3) when holding the other variables constant. *Extraversion* was no significant predictor of the interest in General Practice (*p* = .054). Of the covariates, age and semester significantly predicted the interest in General Practice, and gender did not significantly contribute to the prediction of interest. While an older age was associated with higher interest values, a higher semester was associated with reduced interest in General Practice when controlling for the other variables.

**Table 3:** Effects of the multiple linear regression of the ‘Big five’ personality dimensions on the interest in General Practice

Variables	b	SE	β	t	*p*
Extraversion	0.13	0.07	0.08	1.93	0.054
Agreeableness	0.31	0.07	0.17	4.28	0.000***
Conscientiousness	-0.35	0.10	-0.15	-3.55	0.000***
Neuroticism	0.18	0.07	0.11	2.49	0.013*
Openness	-0.17	0.08	-0.09	-2.23	0.026*
Gender	-0.17	0.13	-0.06	-1.37	0.171
Age	0.05	0.02	0.14	2.66	0.008**
Semester	-0.03	0.01	-0.11	-2.03	0.042*

Note. *b*: regression coefficient, *SE*: standard error, β: standardized regression coefficient, *t*: test; statistic, *p*: significance value; * p < .05, ** p < .01, ***p < .001

### Mediation effects of personality on the intent to currently choose General Practice for specialty

The second model was conceptually mostly equivalent to the linear regression. However,, we added the intent to select General Practice as an outcome variable predicted by the interest in General Practice. The overall model fit was excellent as indicated by a non-significant Chi²-test (χ²(5) = 1.38, *p* = .926). The model fit was also supported by the high comparative fit index (CFI = 1.00), the low root mean square error of approximation (RMSEA = 0.000, 95%-CI: 0.000, 0.018, *p* = .997), and the low standardized root mean square residual (SRMR = 0.000).

Like in the linear regression, *agreeableness*, and *neuroticism* were significant positive predictors of interest in GP, and *conscientiousness* and *openness* were significant negative predictors. Interest in GP was a significant predictor of the intent to select GP. Identically, age was a significant predictor of interest in GP. In addition to these effects that were also found in the linear regression, *extraversion* was a significant predictor of interest. Most indirect effects of the personality dimensions on the intent to select GP mediated by the interest in GP became significant (see Table 3). Only the indirect effect of *extraversion* was on the edge of significance (*p* = .05).

Higher values of *agreeableness* and *neuroticism* predicted a higher possibility of having the intent to select GP mediated by the interest in GP when controlling for the respective other variables. Higher values of *conscientiousness* and *openness* indicated a lower possibility of having the intent to select GP mediated by the interest in GP when controlling for the other variables. A higher age leads to a higher interest when controlling for the other variables.

## Discussion

We demonstrated that students’ personality traits predict their interest in General Practice and their intention to choose GP as a specialty. In addition to personality, higher age was associated with an increased interest in GP. At the same time, interest in GP appeared to be more pronounced in the earlier semesters, while there were no significant gender effects.

### The ‘Big five’

The degree of *agreeableness* most strongly predicted the interest in GP positively and describes interpersonal behaviour: People with higher scores tend to behave in an altruistic, supporting, and compassionate manner. In the GP context, this translates into the ability to establish trustful relationships and to guide patients in a supportive and motivating manner over a long period. Consistently, other studies found higher scores of *agreeableness* in primary care physicians compared to specialists, like surgeons [26, 29].

*Conscientiousness* was the strongest negatively associated factor in the prediction of interest. It refers to one**’**s level of thoughtfulness, goal orientation, and self-control. Individuals with higher scores tend to be more self-disciplined and persistent, while lower scores are associated with preferring spontaneity. In our study, we found lower levels of *conscientiousness* in students with an interest in GP compared to students with other specialty interests. Our results could reflect that students with higher levels of *conscientiousness tend* to choose different, more detail-orientated specialties, like surgery, as Rosenthal et al. found higher scores of conscientiousness in surgeons [38]. Further, our assumption is supported by a study that investigated the preferences for detail-orientated work content among medical students at the time of their transition from medical school to specialist training. It was found that participants considering a career as a General Practitioner tend to reject detail-orientated work content. Instead, they seem to favour a high degree of spontaneity and flexibility to respond quickly to different situations and people [39]. In the GP practice, patients often come unplanned and unscheduled, especially for acute complaints, and wide ranges of clinical pictures have to be diagnosed within a short time [40, 41]. In addition, patients bring their history, personality, and expectations. In this context, flexible and fast responses are important to address the patients’ different needs and to build a trusting doctor-patient relationship [42].

Higher levels of *neuroticism* and lower levels of *openness* influenced the interest in GP. *Neuroticism* refers to relatively stable tendencies to respond with negative emotions to threat, frustration, or loss. Those with low scores tend to exhibit emotional stability and resilience, while those with higher scores are more prone to anxious, stressed, and emotionally driven behaviour. In our study, students with an interest in becoming a GP showed a tendency towards higher scores of *neuroticism* - this goes in the same direction as the results of McCollough et al., who reported higher levels in GPs than in surgeons [43]. Although a higher level of neuroticism is often seen as a challenge due to stress-related strains [44–46], its presence in General Practitioners can be advantageous to some degree. Managing uncertainty is seen as a fundamental aspect of delivering care in General practice [47, 48]. The absence of a definitive diagnosis often leads to concerns among doctors about potentially missing a serious, life-threatening condition among the frequent harmless cases. Research indicates that individuals with higher neuroticism exhibit greater concern about adverse outcomes, which can manifest as heightened vigilance and caution in clinical decision-making [49]. This trait aligns with studies showing that GPs with higher neuroticism levels engage in more diagnostic activities, such as ordering additional tests or investigations [48]. These behaviours reduce the likelihood of missed diagnoses and reflect a commitment to patient safety and thoroughness. Moreover, neuroticism has been positively linked to empathy [50, 51]. Empathy enhances perspective-taking, allowing GPs to better understand and respond to their patients’ emotional and psychological states [52]. This capacity for emotional resonance enables GPs to connect more deeply with patients, fostering trust and open communication and contributing to the sustainability of the doctor-patient relationship.

*Openness* describes an open-mindedness towards new experiences. It is a very broad category, including curiosity, tolerance, interest in art and culture, creativity, and educational experiences, and it is often misunderstood and confused with openness in social contact with other people [53]. In our study, lower levels of *openness* predicted a higher interest in GP. Mullola also observed this result in a study [29]. In this context, lower levels of openness could reflect that these students tend to choose a specialty that is already more familiar to them because they are usually patients of General Practitioners themselves. In line with this, many studies found that early contact and experience in the field of General Practice is a predictor for choosing this specialty in particular [54, 55], and positive role models are shown to influence the choice of General Practice for specialty [56, 57]. As the construct of *openness* encompasses more facets than the other personality factors and its facets are rather loosely related [53], future research should investigate which aspects of *openness* are the most relevant to specialty choices in medical students.

*Extraversion describes* an outward-looking attitude. High levels are associated with the tendency to seek the company of others, enthusiasm, and being receptive to stimulation and excitement. For *extraversion*, a joint contemplation of the effects from linear regression and the path model shows no clear effect: while the linear regression suggests a non-significant effect, the path model reaches the threshold, suggesting a positive association between extraversion and interest in GP. While Bexelius et al. found no differences between the specialties concerning extraversion [26], other studies reported lower levels of extraversion in specialists with no or little patient contact [29, 58] and a positive correlation between extraversion and medical students’ attitudes towards interaction with patients [59]. Given the significant amount of patient interaction and communication involved in their work and the positive impact of extraversion on communication skills [60], the tendency for GPs to exhibit higher levels of extraversion aligns well with their professional demands.

We found relatively small effect sizes for personality dimensions. This is in line with other studies, as Borges and Savickas also reported rather loose associations between personality factors and particular medical specialties [28]. It is not surprising that the effect of personality is modest, given the wide range of other influential factors such as cultural background, regional context, role models, and working conditions, as already outlined in the introduction. Nevertheless, our findings demonstrate a stable influence of personality factors, explaining 7.7% of the variance of the interest in General Practice by the combination of personality dimensions and covariates.

Being older was also associated with an increased interest in GP. General Practice is known to be family-friendly due to the possibility of flexible working models and the lack of (night) shift work, weekend work, or long on-call hours as in hospitals. As they get older, more students are concerned with family planning. Further, some of the students who obtained their university entrance qualification via a commitment to later work as a GP in rural regions (‘Landarztquote’, rural doctor quota ) are older [61].

We found that the interest in GP was higher at the beginning of medical studies when controlling for personality and other covariates, especially age. Teaching in various specialist disciplines and clinical assignments occur later in the curriculum. Consequently, students have limited knowledge of these disciplines early on and do not yet consider them in their decision-making process. Due to low response rates in the higher semesters, it was not yet possible to investigate the change in interest in GP throughout medical studies in our study in more detail.

### Implications for recruiting more GPs

Our study revealed that nearly half of the students were interested in GP, especially in earlier stages of the studies, but with only approximately one in seven students indicating it as their first choice. Therefore, a regular opportunity for reflecting on specialty choices should be provided, because career decisions are very complex and are often made sub-optimally [62]. Counselling should be voluntary, repeatable, and adaptable to changes in student preferences, e.g. due to clinical experiences or significant personal life events, such as becoming a parent.

Supporting students to make decisions that suit their personality can help to increase the number of GPs by enhancing job satisfaction, reducing the risk of job-related burnout, or changes to other specialty areas. Patients also benefit in the form of improved doctor-patient communication and a lower risk of medical malpractice [63]. Next to counselling programmes, voluntary courses for professionally supported self-reflection on personality can help students identify their strengths and potential conflicts. For example, reflecting on high levels of *neuroticism* can reveal a diminished willingness to communicate with patients [49]. Educational interventions in primary care have shown positive effects on trainees’ self-care behaviour, an essential skill for maintaining personal well-being and professional work behaviour [64]. Inspired by this, we included a voluntary course for individual stress management and group sessions of case supervision for our students.

Another contribution to increase the number of GPs should be made at the stage of admission to medical studies. 30–60% of all medical study places in Germany are only allocated to school graduates with the best grades (A-level average 1.0-1.2) [65]. Extremely good school grades correlate strongly with a high level of *conscientiousness*, and it secures a placement at university for younger graduates. Our study suggests that there are fewer of these applicants, but rather older applicants with less *conscientiousness* and higher tolerance who later choose a specialisation in GP. This is why more study places should be allocated specifically via selection procedures where the assessment of personality aspects can be a contributing part of the selection progress. This approach is supported by the results of a study by Knight, which showed a negative influence of dysfunctional personality patterns on academic performance [66]. By assessing all beginner students, it would be possible to target students who have extremely pronounced personality traits that are more likely to lead to difficulties in exam situations, teamwork, or the organisation of the doctor-patient relationship. A study by Ferguson et al. showed that high *conscientiousness* enhanced preclinical knowledge acquisition but reduced the acquisition of clinical knowledge [67]. These students could benefit from interventions such as training to improve their empathy skills or counselling offers to reduce social anxiety or exam anxiety. To sum up, students should be supported in dealing with the challenges of the various stages of their studies, taking into account their personalities within the framework of counselling services.

### Strengths and limitations

A bias in voluntary participation in this survey due to personality factors can be assumed. For instance, Saßenroth found that the chance for ‘eager responding’ in a survey increases with higher levels of *neuroticism* [68]. Furthermore, we could only assess the current choice of GP that might be different from the later choice of specialty. This limitation is compensated by our collected data being representative of the whole cohort of medical students in our faculty according to age and gender, as a comparison of data from the beginners to the 6th year and official data from our Students Deanery shows. Our cohorts will undergo surveys for the next six years, providing ample time to evaluate the medical fields our alumni ultimately pursue. This longitudinal approach may also allow us to address the influences of age and major life events during medical work. In the long term, we might even address the effect of practising as a GP on a GP’s personality. In subsequent studies, we also aim to draw direct comparisons with other disciplines in order to be able to discuss the characteristics of the personality traits between the disciplines. For instance, lower scores of *conscientiousness* in students with an interest in General Practice do not indicate that these students tend to behave carelessly. More likely, there were many students in our sample preferring a more detail-oriented specialty than General Practice. Last, the BFI-K does not specifically address concepts such as resilience or a sense of coherence. However, we were convinced to use the BFI-K to add strength to our study by working with a personality model that has been internationally and widely used to produce well-validated findings on personality [69].

## Conclusion

Our study reveals that students’ personality traits predict their interest in General Practice and their intention to choose it as a specialty. Personality assessments can be integrated into counselling services to help students better understand their traits. Our findings highlight the great potential of considering personality in career counselling during medical education or even of extending admission criteria to medical school by personality-related criteria.

## Electronic supplementary material

Below is the link to the electronic supplementary material.

Supplementary Material 1

Supplementary Material 2

## Data Availability

The raw data supporting the contents of this article are provided on request by the corresponding author.
