# Supplementary material for: Personality and interest in general practice: results from an online survey among medical students
Source: BMC Prim Care. 2024 Dec 12;25:415. doi: 10.1186/s12875-024-02682-0 (PMC11636034; doi:10.1186/s12875-024-02682-0)
Supplement: Supplementary file 1 — Supplementary Material 1 [file 12875_2024_2682_MOESM1_ESM.docx]

Supplement 1: Details on data analysis

To ensure the reliability of the dimensions of the Big Five scales, we calculated Cronbach’s α as a measure for internal consistency (Table X). The values for extraversion were high and acceptable for neuroticism and openness. For agreeableness and conscientiousness, the internal consistency is questionable, but still sufficient. The scales are widely used and are thus retained as such.

*Table X.* Descriptive statistics and Cronbach’s α if applicable

|  | *M* | *SD* | Cronbach's α |
| --- | --- | --- | --- |
| Interest | 3.07 | 1.33 |  |
| Extraversion | 3.73 | 0.83 | .82 |
| Agreeableness | 3.53 | 0.76 | .64 |
| Conscientiousness | 4.12 | 0.56 | .66 |
| Neuroticism | 2.82 | 0.82 | .77 |
| Openness | 3.80 | 0.73 | .71 |
| Age | 26.32 | 4.13 |  |
| Semester | 5.19 | 4.87 |  |

*Note.* *N* = 608. Gender: 70.7 % female

Testing the statistical prerequisites for linear regression

Graphical inspection showed that homoscedasticity was given while the residues showed some slight deviation from normal distribution which still seemed acceptable to us (bimodal distribution). Cook’s distance and standardized DF_BETA_ (all < 1) indicated no influential cases that might distort the results of the linear regression. The predictor variables did not show multicollinearity (Table X1, all tolerances > .1).

Table X1. Tolerances for the multiple linear regression on the interest in GP.

|  | Tolerance |
| --- | --- |
| Extraversion | 0.87 |
| Agreeableness | 0.93 |
| Conscientiousness | 0.90 |
| Neuroticism | 0.82 |
| Openness | 0.92 |
| Gender | 0.84 |
| Age | 0.53 |
| Semester | 0.55 |

Table X2. Estimations of the path model including direct effects, thresholds, and indirect effects

| Effects | *b* | 95%-CI | *SE* | β | *z* | *p* |
| --- | --- | --- | --- | --- | --- | --- |
| Extraversion -> Interest | 0,14 | (0,00, 0,27) | 0,07 | 0,08 | 1,97 | 0,049 |
| Agreeableness -> Interest | 0,3 | (0,15, 0,45) | 0,08 | 0,17 | 3,85 | 0 |
| Conscientiousness -> Interest | -0,33 | (-0,54, -0,11) | 0,11 | -0,14 | -2,98 | 0,003 |
| Neuroticism -> Interest | 0,17 | (0,03, 0,31) | 0,07 | 0,11 | 2,42 | 0,015 |
| Openness -> Interest | -0,17 | (-0,32, -0,01) | 0,08 | -0,09 | -2,09 | 0,037 |
| Gender -> Interest | -0,15 | (-0,40, 0,10) | 0,13 | -0,05 | -1,19 | 0,235 |
| Age -> Interest | 0,05 | (0,01, 0,08) | 0,02 | 0,15 | 2,62 | 0,009 |
| Semester -> Interest | -0,03 | (-0,06, 0,00) | 0,01 | -0,1 | -1,93 | 0,054 |
| Interest -> Intention | 0,65 | (0,58, 0,71) | 0,03 | 0,84 | 19,36 | 0 |
| Gender -> Intention | 0,07 | (-0,24, 0,38) | 0,16 | 0,03 | 0,45 | 0,654 |
| Age -> Intention | 0,03 | (-0,01, 0,06) | 0,02 | 0,1 | 1,34 | 0,182 |
| Semester -> Intention | -0,02 | (-0,05, 0,01) | 0,02 | -0,11 | -1,56 | 0,12 |
| threshold Intention | 3,99 | (2,34, 5,64) | 0,84 | 3,85 | 4,75 | 0 |
| Extraversion -> Interest -> Intention | 0,09 | (0,00, 0,18) | 0,04 | 0,07 | 1,96 | 0,05 |
| Agreeableness -> Interest -> Intention | 0,19 | (0,09, 0,29) | 0,05 | 0,14 | 3,82 | 0 |
| Conscientiousness -> Interest -> Intention | -0,21 | (-0,35, -0,07) | 0,07 | -0,11 | -2,98 | 0,003 |
| Neuroticism -> Interest -> Intention | 0,11 | (0,02, 0,20) | 0,05 | 0,09 | 2,41 | 0,016 |
| Openness -> Interest -> Intention | -0,11 | (-0,21, -0,01) | 0,05 | -0,08 | -2,08 | 0,037 |
