# Supplementary material for: Personality and interest in general practice: results from an online survey among medical students
Source: BMC Prim Care. 2024 Dec 12;25:415. doi: 10.1186/s12875-024-02682-0 (PMC11636034; doi:10.1186/s12875-024-02682-0)
Supplement: Supplementary file 2 — Supplementary Material 2 [file 12875_2024_2682_MOESM2_ESM.docx]

Invitation letter for survey (email)

Original German wording (please, see below for English translation):

Liebe Studierende,

mit dem Start Ihres Studiums laden wir Sie ganz herzlich dazu ein, an unserer spannenden und lohnenden Befragung zur Studienwahl und Facharztorientierung teilzunehmen. Unter den Teilnehmenden verlosen wir **originelle Tassen** **mit Fakultätslogo:**


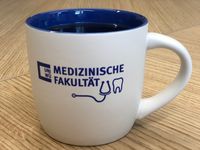


Machen Sie also unbedingt mit! Hier finden Sie den **Link zur Umfrage**: [DIRECT_ONLINE_LINK]

Die Bearbeitungsdauer beträgt ca. **15-30 Minuten** und ist in der Regel durch **einfaches Anklicken** sowohl auf mobilen Geräten als auch mit Unterbrechung (durch Zwischenspeichern) durchführbar. Die Umfrage wird mittels des elektronischen Befragungssystems EvaSys® durchgeführt. Alle von Ihnen gemachten Angaben sind **anonym und freiwillig**. Wir bedanken uns sehr, dass Sie sich für unsere Umfrage Zeit nehmen!

Neben der Möglichkeit Ihre Einstellung zum Studium zu reflektieren, können Sie einen Blick in die Zukunft werfen und Aspekte der ärztlichen Tätigkeit für sich persönlich einschätzen. So erfahren Sie auch etwas über sich selbst.

Durch Ihre Teilnahme ermöglichen Sie es uns, den Zusammenhang zwischen Zugang zum Studium, Studienverlauf, persönlichen Einstellungen und der späteren Facharztwahl näher zu untersuchen. Die Ergebnisse werden uns helfen, Zugangswege zu optimieren und das Studienangebot entsprechend anzupassen, sowie Fördermöglichkeiten auszubauen. Zudem erhalten wir ein aktuelles Stimmungsbild.

Nach erfolgreicher Teilnahme erhalten Sie eine **Teilnahmebescheinigung** (sowohl im Browser, als auch per E-Mail). Bewahren Sie diese auf, damit Sie (im Falle der Auslosung) Ihre Tasse nach Abschluss der Befragung abholen können. Dafür erhalten Sie automatisch eine Benachrichtigung per E-Mail.

Die Teilnahme an der Befragung ist **bis Ende November 2023** möglich.

Bei Rückfragen stehen wir gerne zur Verfügung.

**Vielen Dank für Ihre Mitwirkung!**

**English translation**

Dear students,

With the start of your studies, we cordially invite you to take part in our exciting and rewarding survey on your choice of study and specialist orientation. Among the participants, we will give away **original mugs with the faculty logo**:


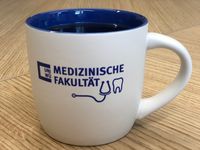


So be sure to participate! Here you will find the **link to the survey**: [DIRECT_ONLINE_LINK]

The processing time is approx. **15-30 minutes** and can usually be done with a **simple click** on mobile devices as well as with an interruption (by caching). The survey is conducted using the electronic survey system EvaSys®. All information you provide is **anonymous and voluntary**. Thank you very much for taking the time for our survey!

In addition to the opportunity to reflect on your attitude to your studies, you will be able to look into the future and assess aspects of the medical profession for yourself. This way you will also learn something about yourself.

Your participation enables us to examine the relationship between access to studies, course of studies, personal attitudes and the subsequent choice of specialist. The results will help us to optimise access routes and adapt the study offer accordingly, as well as expand funding opportunities. We also get an up-to-date picture of the mood.

After successful participation, you will receive a **certificate of participation** (both in the browser and by e-mail). Keep it so that you can collect your cup (in the event of a draw) after the survey has been completed. You will automatically receive a notification by e-mail.

Participation in the survey is possible until the **end of November 2023.**

If you have any questions, we are happy to assist you.

**Thank you very much for your cooperation!**
